# Flavonoid Rutin Reduces Intestinal Inflammation in an Experimental Model of Parkinson's Disease

**DOI:** 10.1007/s12640-026-00789-z

**Published:** 2026-03-28

**Authors:** Livia Bacelar De Jesus, Annyta Fernandes Frota, Fillipe Mendes De Araújo, Fabine Correia Passos, Nestor Adrian Guerrero Gutierrez, Luana Brunelly Araujo de Lima, Victor Diogenes Amaral Silva, Gyselle Chrystina Baccan, Marcelo Biondaro Gois, Silvia Lima Costa

**Affiliations:** 1 Laboratory of Neurochemistry and Cellular Biology, Institute of Health Sciences, Federal University of Bahia, Av. Reitor Miguel Calmon S/N, Salvador, 40231-300 Brazil; 2 Center for Biological and Health Sciences, Federal University of Western Bahia, R. da Prainha, N. 1326, Barreiras, 47810-047 Brazil; 3 Laboratory of Neuroendocrine-Immunology, Federal University of Bahia, Av. Reitor Miguel Calmon S/N, Salvador, 40231-300 Brazil; 4 Health Sciences Center, Federal University of Reconcavo da Bahia, Av. Carlos Amaral, 1015, Santo Antônio de Jesus, 44570-000 Brazil; 5 Faculty of Health Sciences, Federal University of Rondonópolis, Av. dos Estudantes, 5055, Cidade Universitária, Rondonópolis, 78736-900 Brazil

**Keywords:** Rutin, Enteric nervous system, Gut microbiota

## Abstract

The enteric nervous system (ENS), a complex network of neurons and glial cells, is essential for maintaining intestinal homeostasis and is implicated in neurodegenerative diseases such as Parkinson's disease (PD). The gut-brain axis, modulated by gut microbiota (GM), is influenced by dietary compounds that can alter its composition. Despite advances in the understanding of PD pathophysiology, effective treatments remain limited, underscoring the need for novel therapeutic approaches. Among plant-derived compounds, the flavonoid rutin has shown significant antioxidant, anti-inflammatory, and neuroprotective properties in vivo. This study evaluated the effects of rutin on leukocyte infiltration, intestinal morphology, and GM composition in an experimental model of PD. Adult male Wistar rats received a stereotaxic injection of 6-hydroxydopamine (6-OHDA) and were treated orally with rutin (10 mg/kg) for 14 days. Intestinal segments were analyzed histomorphometrically, and fecal samples were assessed for the abundance of Firmicutes, Bacteroidetes, Prevotellacea, Entererobactereacea, *Bifidobacterium* sp and *Lactobacillus* sp. by PCR. Rutin administration significantly reduced intraepithelial lymphocyte infiltration and goblet cell numbers in the ileum and colon and prevented hyperplasia of Paneth cells in the ileum. Importantly, GM composition remained unchanged following rutin treatment. These findings demonstrated that rutin reduces intestinal inflammation in PD models without altering gut microbiota composition, highlighting its potential as a therapeutic strategy.

## Introduction

The enteric nervous system (ENS) is an extensive reflex control system for digestive function that controls, in conjunction with the central nervous system (CNS), patterns of contractile activity, local blood flow, and transmucosal fluid movement (Wood 2018). Anatomically, ENS is composed of neurons and enteric glial cells organized into interconnected ganglia that extend from the distal portion of the esophagus to the end of the large intestine. Together, these cells control and coordinate intestinal motility and secretion, ensuring the functional integration of the gastrointestinal tract (Holland et al. 2021; Furness et al. 2014).

Studies have shown that enteric glial cells play an important role in different neurodegenerative disorders such as Parkinson's disease (PD) which can, in some cases, start in the enteric nervous system and spread through the vagal nerve to the brain stem (Furness et al. 2014; Luesma et al. 2024). PD is the second most important neurodegenerative disease after Alzheimer's disease and is mainly characterized by the progressive loss of dopaminergic neurons in the substantia nigra pars compacta, generating motor dysfunctions (bradykinesia, rigidity, postural instability and resting tremor) and non-motor symptoms including gastrointestinal dysfunction (Shannon and Vanden Berghe 2018; Herrero and Morelli 2017).

The importance of the gut-brain axis in maintaining homeostasis has long been considered. However, the emergence of the gut microbiota (GM) as a major regulator of gut-brain function has led to an appreciation of the importance of a distinct microbiota-gut-brain axis. The microbiota and the brain communicate with each other through several routes, including the immune system, tryptophan metabolism, the vagus nerve, and the enteric nervous system (Cryan et al. 2019; Sampson et al. 2016). The composition of human GM is dynamic and varies with age. Factors such as environmental stressors, use of antibiotic drugs, chemotherapy and radiotherapy treatments, geographic location, genetics and nature of nutritional provision influence the diversity of IM (Góralczyk-Bińkowska et al. 2022).

Studies highlight that different types of diet affect the composition of GM and the production of microbial metabolites (Berding et al. 2021; Tomova et al. 2019). In this sense, flavonoids, compounds originating from the secondary metabolism of plants, are sources of several molecules with biological potential, including antioxidant, antitumor, neuroprotective, anti-inflammatory and antimicrobial activities (Spagnuolo et al. 2018). The flavonoid rutin (3,3',4',5,7 pentahydroxyflavone-3 rutinoside) belongs to the flavonol classis and present in large quantities in pods of the plant *Dimorphandra mollis* (Fabaceae), popularly known as “faveira”, “favela” and “fava d'anta”, a plant widely distributed in the Cerrado of Brazil, where it is native and occurs mainly in the states of Mato Grosso, Goiás, Minas Gerais and São Paulo, as well as in Bahia. Preclinical studies have demonstrated that rutin presents highly antioxidant properties that have been associated with anti-inflammatory effects, diminishing the expression of pro-inflammatory mediators like tumor necrosis factor-α, interleukin (IL)−6, cyclooxygenase-2, and IL-1β (Muvhulawa et al. 2022). Moreover, rutin and its derivatives appear to mitigate the effects of aging on the brain. In a previous study, we demonstrated the neuroprotective effect of rutin in organotypic brain cultures subjected to excitotoxic damage with excess of neurotransmitter glutamate (Ferreira et al. 2021).

On the other hand, in a more recent study using intracerebral injections of endogenous neurotoxin aminochrome or 6-hydroxydopamine (6-OHDA) as an in vivo model of PD, the neuroprotective effect of the flavonoid rutin administered orally was characterized, preventing the loss of dopaminergic neurons in the substantia nigra pars compacta (SNPc) in addition to reducing microgliosis and astrogliosis and the mRNA levels of interleukin-1β (IL-1β) and increasing the mRNA levels of nerve-derived neurotrophic factor (NGF) and glial-derived neurotrophic factor (GDNF) (De Araújo et al. 2023).

Furthermore, in an in vivo model of PD induced by 6-OHDA we also showed that rats treated orally with rutin (10 mg/kg) presented an improvement in motor capacity and intestinal transit, without interfering with the cell population; rutin revealed modulatory activities in the myenteric plexus, providing relevant answers regarding the effect of the flavonoid in this system and the potential application of adjuvant treatment for PD (Jesus et al. 2024). However, the effects of the flavonoid in microbiota and in the enteric tissue integrity and inflammatory status in the PD model condition were not yet characterized. Thus, in the present study we evaluated the effect of the flavonoid rutin on leukocyte infiltration, intestinal morphology, and GM composition in an experimental model of PD.

## Results

### 6-OHDA Induced Loss of Dopaminergic Neurons in the Rat SNpc

**Fig. 1:**
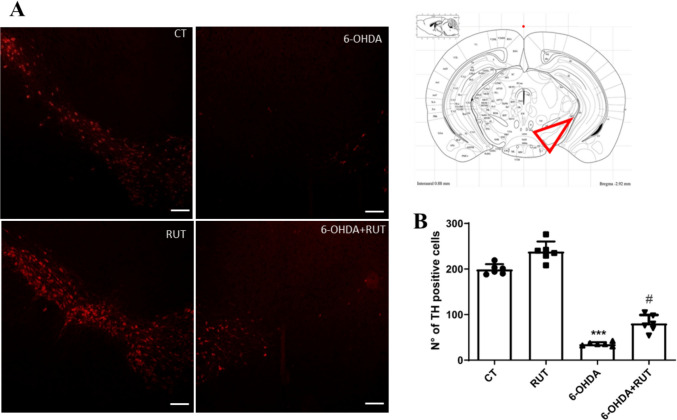
6-hydroxydopamine (6-OHDA) induces dopaminergic degeneration in Substance Nigra pars compacta. **A** Representative images of immunofluorescence for tyrosine-hydroxilase positive neurons (red, TH +). Control group (CT): animals injected with saline solution without treatment (*n* = 6); Group 6-OHDA: animals injected with 6-OHDA without treatment (*n* = 6); Group RUT: animals injected with saline and treated with rutin (*n* = 6); Group 6-OHDA + RUT: animals injected with 6-OHDA and treated with rutin (*n* = 6); (× 5 magnification, scale bar = 200 µm). **B** Quantification of TH + cells in the SNpc of rats. The reduction in the number of dopaminergic neurons in 6-OHDA when compared to Control group (CT, ***) and the inhibition of the effect of 6-OHDA by rutin in the RUT + 6-OHDA group (when compared to the 6-OHDA group (#). Data are represented by means ± S.D. Statistical analysis was performed using the one − way ANOVA parametric analysis test, followed by post hoc Tukey’s multiple comparison test *p* < 0.001

6-OHDA is a selective catecholaminergic neurotoxin, widely used to model Parkinson’s disease. Intrastriatal injection of 6-OHDA induces a rapid loss of tyrosine hydroxylase (TH) immunoreactivity in the striatum, which reaches maximum levels. To evaluate the effect of 6-OHDA on dopaminergic neuron degeneration, an immunofluorescence assay for TH was performed in the substantia nigra pars compacta (SNpc) of the rats brains (Fig. 1). A significant reduction in the number of TH-positive (TH +) neurons in the SNpc of rats from the positive control group (6-OHDA) (18.00 ± 2.483%, *p* < 0.0001) compared to the negative control group (CT) animals injected with saline solution without treatment (200.0 ± 7.153%) was observed. These results demonstrate that 6-OHDA injections effectively induced degeneration of dopaminergic neurons in this experimental model.

## Striatal Injection of 6-OHDA in a Neuron Model of Dopaminergic Degeneration Affected the Intestinal Wall of Rats

Histomorphometric analysis of the intestinal wall revealed a significant increase in the thickness of the ileal musculature in the 6-OHDA group (66.64 ± 5.32) when compared to the control group (54.30 ± 12.45) (Table 1). However, we observed a reduction in the thickness of the submucosa of the animals submitted to the injection of 6-OHDA and treated with rutin (6-OHDA + RUT) (28.02 ± 3.96) both in comparison to the control group (34.60 ± 3.713) and compared to the control group (34.60 ± 3.713). to the 6-OHDA group (33.86 ± 3.92). A significant reduction in the width of the villi of this segment was also observed in the animals of the 6-OHDA + RUT group (65.46 ± 17.46) when compared to the animals of the 6-OHDA group (79.79 ± 9.57). Regarding the colonic segment, a significant reduction in mucosal thickness was observed in the 6-OHDA group (196.0 ± 30.55) compared to the control (265.1 ± 49.38), as well as a reduction in the depth of the crypt both in the 6-OHDA (121.9 ± 30.77) and for the 6-OHDA + RUT group (110.07 ± 33.93) when compared to the control group (181.1 ± 36.88). The crypts present in the colon of animals in the 6-OHDA + RUT group (32.54 ± 7.59) also showed a reduction when compared to the control (42.31 ± 4.22).

**Table 1:** Histomorphometric analysis of the strata that make up the wall of the ileum and colon

		ILEUM			
		Control	RUT	6OHDA	6OHDA + RUT
Total wall		319,9 ± 37,10	318,3 ± 61,72	360,7 ± 75,29	343,3 ± 97,82
Muscular	Thickness	54,30 ± 12,45	50,33 ± 6,95	66,64 ± 5,32**	57,83 ± 4,25
Submucosa		34,60 ± 3,713	37,91 ± 4,89	33,86 ± 3,92	28,02 ± 3,96**#
Mucosa		269,8 ± 74,83	286,9 ± 51,73	323,2 ± 74 ± 81	307,9 ± 79,80
Crypts	Depth	83,77 ± 14,15	87,91 ± 15,69	93,51 ± 13,02	83,24 ± 14,19
	Width	64,54 ± 8,08	82,12 ± 15,50*	64,88 ± 9,49	63,46 ± 13,47
Vilos	Height	163,9 ± 46,19	160,10 ± 46,60	166,31 ± 54,52	181,3 ± 54,53
	Width	74,55 ± 4,36	79,93 ± 8,79	79,79 ± 9,57	**65,46 ± 17,46#**
		COLON			
		Control	RUT	6OHDA	6OHDA + RUT
Total wall		485,0 ± 53,09	485,7 ± 54,34	480,4 ± 59,29	486,1 ± 56,38
Muscular	Thickness	179,1 ± 29,29	191,9 ± 34,44	157,8 ± 40,24	184,4 ± 39,54
Submucosa		34,38 ± 11,19	38,75 ± 7,82	35,91 ± 7,24	36,45 ± 8,66
Mucosa		265,1 ± 49,38	272,0 ± 44,92	196,0 ± 30,55*	238,3 ± 57,61
Crypts	Depth	181,1 ± 36,88	180,7 ± 30,46	121,9 ± 30,77***	110,07 ± 33,93***
	Thickness	42,31 ± 4,22	41,36 ± 3,69	42,00 ± 3,86	32,54 ± 7,59***#

**Fig. 2:**
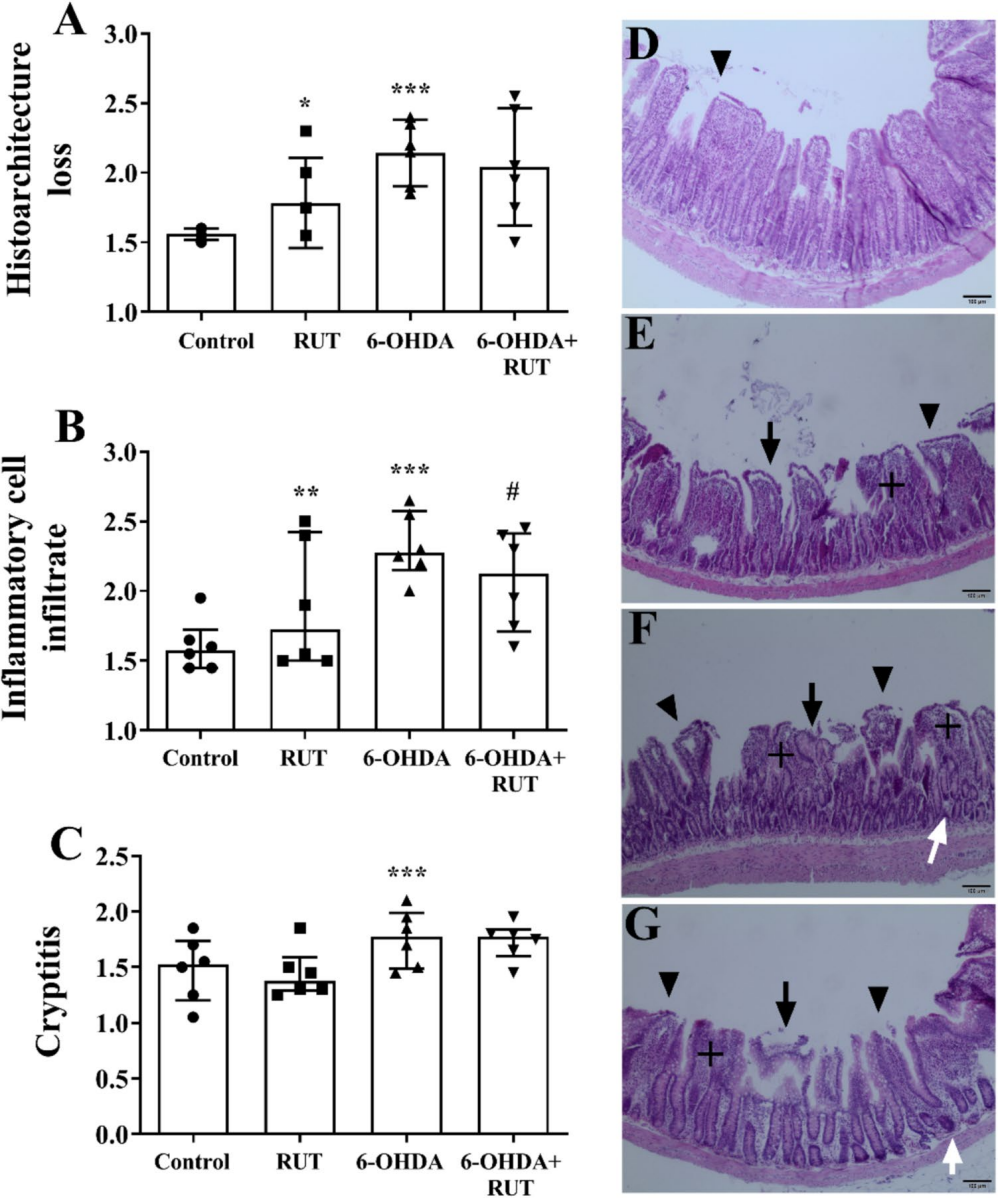
Semi-quantitative histopathological evaluation (score) performed on strata that make up the ileum wall of rats treated or not with 10 mg/Kg rutin and divided into Control, RUT, 6-OHDA or 6-OHDA + RUT groups. **A** Changes in the histoarchitecture of the ileum mucosa; **B** presence and distribution of inflammatory infiltrates in the ileum wall; **C** Inflammation in the intestinal crypts. Data are expressed as median ± interquartile range. Statistical analysis was performed using the Kruskal–Wallis test followed by Dunnett’s post-test, as data showed a non-parametric distribution (D’Agostino–Pearson test). *p < 0.05, **p < 0.01, **p < *0.001 vs. Control; #p* < *0.05 vs. 6-OHDA*. **D**-**G** Representative photomicrographs of the ileum wall. **D** Mild hyperplasia with slightly altered mucosa and discrete diffuse inflammatory cell infiltrate restricted to the lamina propria (arrowhead). **E** Marked hyperplasia (+), erosion of the epithelial surface, flattening (atrophy) of the mucosa and moderate blunting and fusion of villi (arrowhead), severe diffuse inflammatory cell infiltrate in the mucosa (arrow). **F** and **G** Marked hyperplasia and loss of goblet cells (+), erosion of the epithelial surface, flattening (atrophy) of the mucosa and moderate blunting and enlargement of the villi (arrow), severe multifocal to diffuse transmural inflammatory cell infiltrate (arrowhead), and neutrophils dispersed between crypt epithelial cells (white arrow). Staining in H&E; 10 × objective; 100 µm bar

**Fig. 3:**
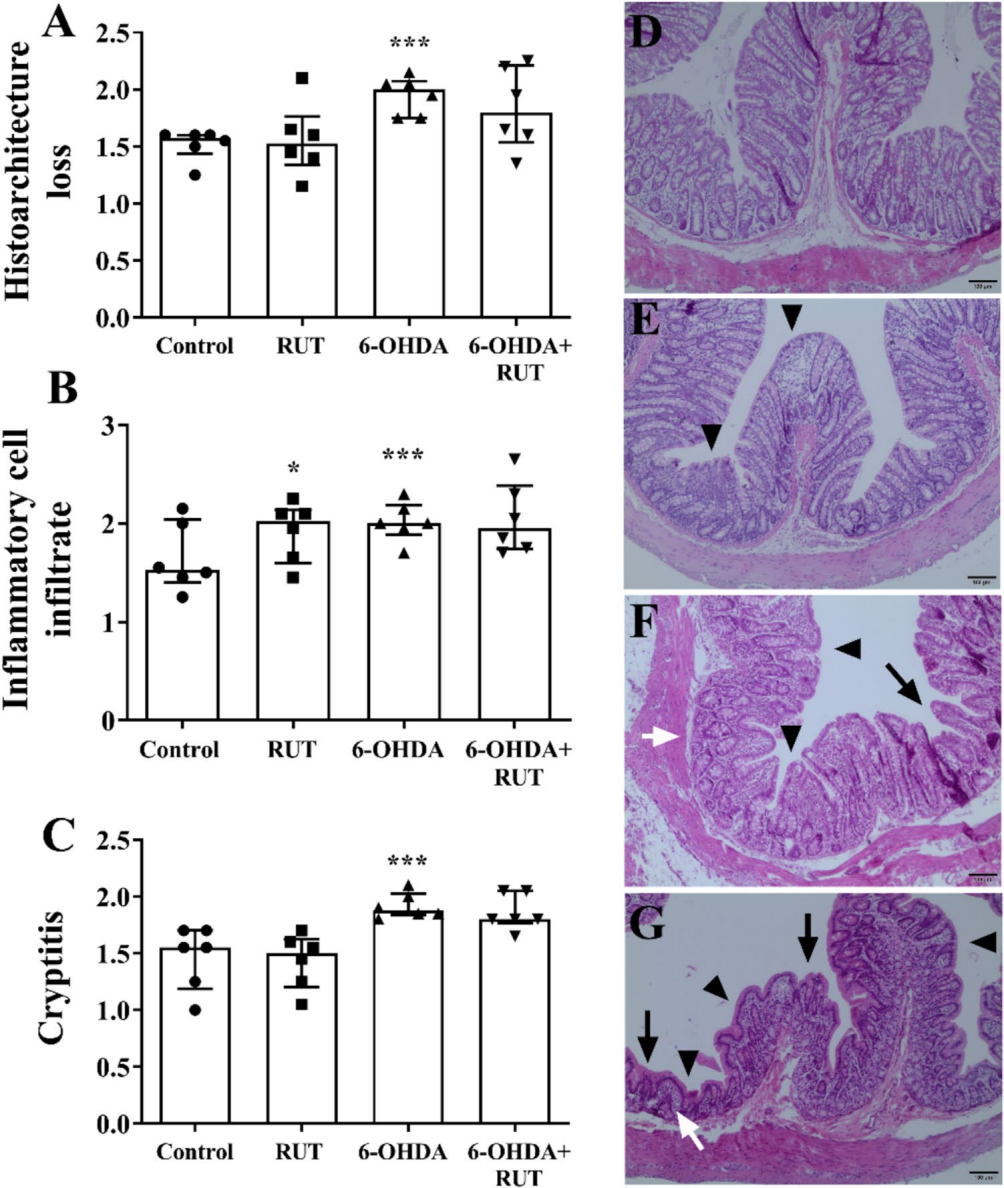
Histopathological analysis of strata that make up the colon wall. Semiquantitative histopathological evaluation (score) performed on strata that make up the colon wall of rats treated or not with 10 mg/Kg rutin and distributed into Control, RUT, 6-OHDA or 6-OHDA + RUT groups. **A** Changes in the histoarchitecture of the colonic mucosa; **B** presence and distribution of inflammatory infiltrates in the colon wall; **C** Inflammation in the intestinal crypts. Data were compared using One-way ANOVA followed by Kruskal–Wallis test and Dunnett's post-test and presented as median ± interquartile deviation (*n* = 6). * *P* < 0.05, ** *P* < 0.01 or *** *P* < 0.001 compared to Control. **D**-**G** Representative photomicrographs of the colon wall. **D** Normal mucosa, intact submucosal mesh, normal muscular layer and absence of inflammatory cell infiltrate. **E** Mild hyperplasia and discrete focal to multifocal inflammatory cell infiltrate restricted to the lamina propria (arrowhead). **F** Moderate hyperplasia, loss of goblet cells and flattening (atrophy) of the mucosa (arrow), moderate multifocal to diffuse inflammatory cell infiltrate (arrowhead), and neutrophils scattered among the crypt epithelial cells (white arrow). **G** Marked hyperplasia and loss of goblet cells, erosion of the epithelial surface and flattening (atrophy) of the mucosa (arrow), severe multifocal to diffuse transmural inflammatory cell infiltrate (arrowhead), and erosion and abscess with dispersed neutrophils among the epithelial cells and in the crypt lumen (white arrow). Staining in H&E; 10 × objective; 100 µm bar

Histopathological evaluation was performed in the strata that make up the ileum and colon wall of the animals (Figs. 2 and 3). Changes in the mucosal histoarchitecture (Figs. 2A and 3A) of the 6-OHDA and 6-OHDA + RUT groups were observed when compared to the control group (*p* < 0.001), as well as an increase in the number and distribution of inflammatory infiltrates (Figs. 2B and 3B) (*p* < 0.001), in addition to the presence of inflammation in the intestinal crypts, (*p* < 0.001) compared to the control (Figs. 2C and 3C), both in the ileal segment and in the colonic segment. A subtle increase in the inflammatory infiltrate was also observed in the ileum and colon wall of animals treated only with rutin. In animals injured with 6-OHDA that were treated with rutin, we observed a reduction in the inflammatory infiltrate when compared to the group injured by 6-OHDA without treatment (*p* < 0.05) (Figs. 2B and 3B).

Histomorphometric analysis of the strata that make up the wall of the ileum and colon of animals injected with 21 μg of 6-hydroxydopamine (6-OHDA) or saline (Control) in the striatum, treated or not with rutin (RUT, 10 mg/Kg), injected with 6-OHDA (positive control) and treated with rutin (6-OHDA + RUT).

### Rutin Treatment Reduced the Number of Intraepithelial Lymphocytes (IELs) in Rats Injected with 6-OHDA

**Fig. 4:**
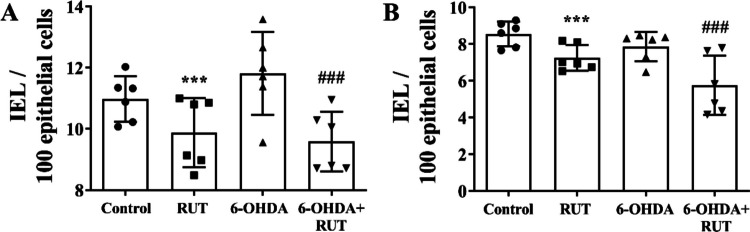
Quantification of intraepithelial lymphocytes (IELs). **A** Number of IELs per 100 epithelial cells in the ileal mucosa and **B** Number of IELs per 100 epithelial cells in the colonic mucosa of rats injected with 21 μg of 6-OHDA or saline in the striatum, treated or not with rutin (10 mg/Kg) distributed in Control, RUT, 6-OHDA or 6-OHDA + RUT groups. Data were compared using One-way ANOVA followed by Tukey's post-test and presented as mean ± standard deviation. * *P* < 0.05 and *** *P* < 0.001 compared to control; ### *P* < 0.001 compared to the 6-OHDA group

The number of IELs was measured in the ileum (Fig. 4A) and colon (Fig. 4B) segments. In the ileum, a reduction in IEL count was observed in the RUT group compared to the control (*p* < 0.01) and in the 6-OHDA + RUT group compared to the 6-OHDA group (*p* < 0.001). In contrast, an increase in the number of IELs per 100 epithelial cells was observed in the 6-OHDA group compared to the control (*p* < 0.05). In the colon (Fig. 4B), a similar reduction in IEL count was found in the RUT group compared to the control (*p* < 0.01), as well as in the 6-OHDA + RUT group compared to the 6-OHDA group (*p* < 0.001). No significant changes were observed in the 6-OHDA group.

### Treatment with Rutin Induced Hyperplasia and Proliferation of Paneth Cells in Ileal segment of Rats Injected with 6-OHDA

**Fig. 5:**
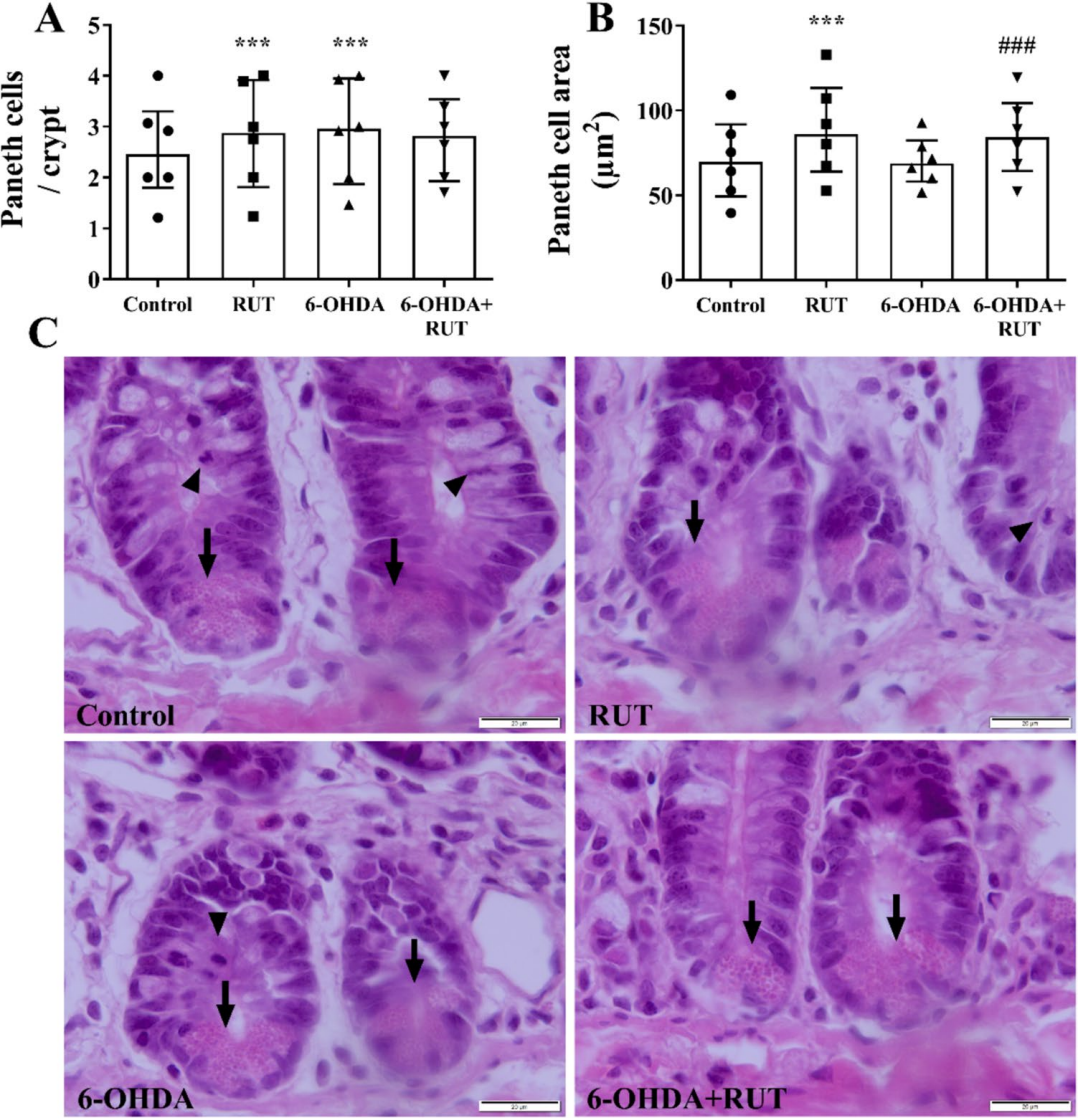
Quantification of Paneth cells in Ileum. In **A** number of Paneth cells per intestinal crypt and **B** profile of areas (µm^2^) of Paneth cells in the mucosa of the ileum of rats injected with 21 μg of 6-OHDA or saline in the striatum, treated or not with rutin (10 mg/Kg) distributed in Control, RUT, 6-OHDA or 6-OHDA + RUT groups. Data were compared using One-way ANOVA followed by Kruskal–Wallis test and Dunnett's post-test and presented as median ± interquartile deviation. *** *P* < 0.001 compared to Control; ### *P* < 0.001 compared to the 6-OHDA group. **C** Representative photomicrographs of H&E-stained sections illustrate Paneth cells at the base of intestinal crypts (arrows) in the mucosa of the ileum of rats. Note the frequent presence of mitotic figures (arrowhead). 40 × objective; 20 µm bar

We evaluated the distribution of Paneth cells in crypts of the ileum of animals injected with 6-OHDA. A significant increase in these cells was observed in the ileal segment of animals in the RUT and 6-OHDA + RUT groups, as shown in Fig. 5. Both, the quantity (Fig. 5A) and the size (Fig. 5B) of these cells were increased in the RUT and 6-OHDA groups (*p* < 0.001), when compared with the control group.

### Striatal Injection of 6-OHDA Reduced Goblet Cells in Rat Ileum and Colon

**Fig. 6:**
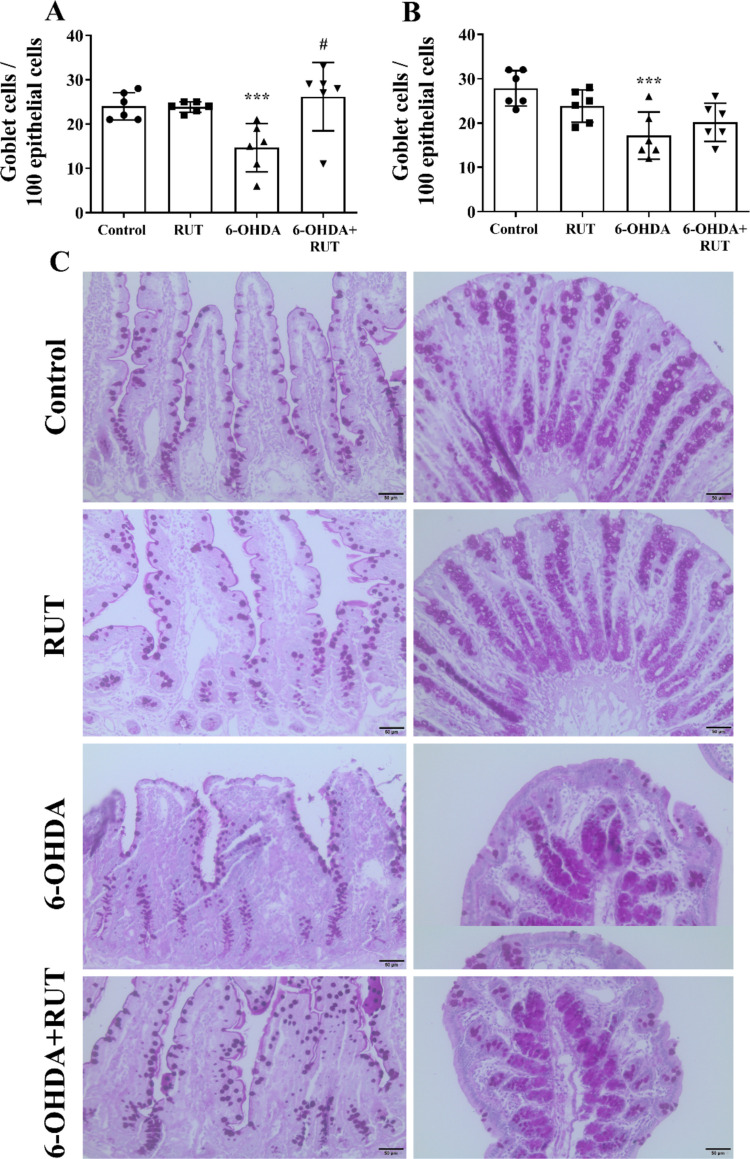
Quantification of goblet cells. **A** Number of goblet cells per 100 epithelial cells in the ileal mucosa **A**, colon **B**; Rats injected with 21 μg of 6-OHDA or saline in the striatum, treated or not with rutin (10 mg/Kg) distributed in Control, RUT, 6-OHDA or 6-OHDA + RUT groups. Data were compared using One-way ANOVA followed by Tukey's post-test and presented as mean ± standard deviation (*n* = 6). * *P* < 0.05 or ** *P* < 0.01 compared to Control; # *P* < 0.05 compared to 6-OHDA. **C** Representative photomicrographs of PAS-stained sections illustrate goblet cells in the intestinal epithelium. 20 × objective; 50 µm bar

A significant reduction in the number of goblet cells in the ileum and colon of the animals of the 6-OHDA group was observed when compared to the control (Fig. 6). In the ileal segment (Fig. 6A), a reduction was observed in the 6-OHDA group (*p* < 0.001) whereas in the 6-OHDA + RUT group a reduction was observed both when compared to the control group and to the 6-OHDA group (*p* < 0.05) (Fig. 6B).

### Gut Microbiota was not Altered in Animals Treated with Ruti

**Fig. 7:**
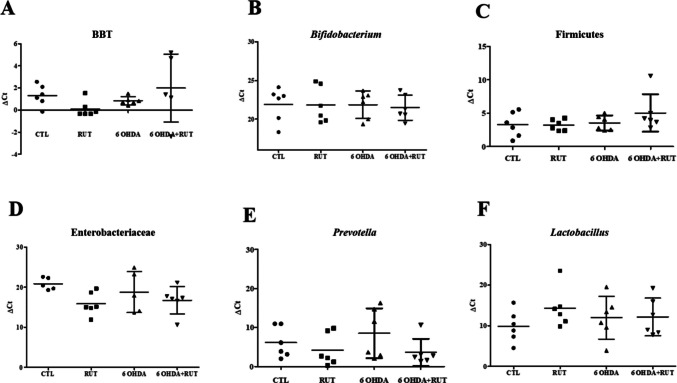
Composition of the intestinal microbiota. Rats injected with 21 μg of 6-OHDA or saline in the striatum, treated or not with rutin (10 mg/Kg) distributed into groups Control (*n* = 6), RUT (*n* = 6), 6-OHDA (*n* = 6) and 6-OHDA + RUT (*n* = 6). Values are presented in 2^-(∆Ct) for the relative abundance of the evaluated bacteria. **A** Bacteria total population of the intestine (BTP). **B** Relative abundance for bacteria of the genus *Bifidobacterium*. **C** Relative abundance of the phylum Firmicutes. **D** Relative abundance of the family Enterobacteriaceae. **E** Relative abundance of the genus *Prevotella*. **F** Relative abundance of the genus *Lactobacillus.* The difference between the groups was evaluated using the Student's T test

The relative abundance of bacteria, represented in 2^-(∆Ct) (Fig. 7), was obtained for bacteria from the phylum Bacteroidetes (BTT) (Fig. 7A), the genus *Bifidobacterium* (Fig. 7B), the phylum Firmicutes (Fig. 7C), from the phylum Enterobacteria (Fig. 7D), from the genus *Prevotella* (Fig. 7E), and the genus *Lactobacillus* (Fig. 7F). The total number of intestinal bacteria in each animal was also determined and used as an endogenous control. No significant difference was observed between the groups in these parameters.

## Methods

### Animals

The study was carried out with 24 male Wistar rats (3 months old, weight 250–300 g) from the Animal Facility of the Laboratory of Neuroscience in the Institute of Health Sciences located at the Federal University of Bahia and kept in an isolated room with access to water and food (Nuvilab CR-1 irradiated) ad libitum, at 20 ± 2 °C, relative humidity (45–55%), light 12:12 h cycle) and with 20 air changes per hour. The local Institutional Review Board for Animal Experimentation approved all procedures involving animals (Health Sciences Institute of the Federal University of Bahia) (CEUA-ICS/UFBA 011/2017). All experiments involving animals were conducted in accordance with the guidelines established by the Brazilian College of Animal Experimentation (COBEA) and the National Council for the Control of Animal Experimentation (CONCEA).

The experiment was conducted in blind, controlled, and randomized test formats. All animals were submitted to stereotaxic surgery and were divided into four groups: control rats that received only saline orally (Control, *n* = 6); rats that were treated with flavonoid rutin (10 mg/kg) (RUT, *n* = 6); rats received intracerebral injection of 21 μg of 6-hydroxydopamine (6-OHDA, *n* = 6); and rats received intracerebral injection of 6-OHDA and were treated with rutin (6-OHDA + RUT, *n* = 6). The animals were exposed orally by gavage using a large, curved needle for rats made of stainless steel manufactured by AD Surgical Instruments, treated with the flavonoid rutin (10 mg/kg) obtained from Sigma-Aldrich with 98% purity diluted in a 0.5% carboxymethylcellulose (CMC) vehicle for 14 days. On the 15th day after 6-OHDA injection, all rats were euthanised by deep anaesthesia with ketamine hydrochloride 100 mg/kg and xylazine hydrochloride 14 mg/kg by intraperitoneal injection and perfused transcardially with 0.1 M PBS, and the biological material was collected.

### Parkinson’s Diseases Model

For the induction of PD was injected 21 μg of 6-OHDA (Sigma-Aldrich) diluted in 6 μL of 0.9% saline with 0.2% ascorbic acid in three different points into the right striatum (2 μg/μL of 6-OHDA in each point). Rats were anesthetized with ketamine and xylazine (80 mg/Kg; 10 mg/Kg, i.p.) under stereotaxic conditions. The injection was performed at the following coordinates: 2.5 mm mediolateral (ML) and 5 mm dorsoventral (DV) (first point), and 3 mm ML, 0.5 mm anteroposterior (AP) and 6 mm DV (second point) and 3.7 mm mediolateral (ML) and 0.9 mm anteroposterior (AP) and 6.5 mm dorsoventral (DV) (third point) (Paxinos and Watson, 2005). In the control and rutin groups 2 μL of saline (0.9%) was injected in three different points into the right striatum during 5 min. At the end of injection, the needle was held in place for an additional 5 min to avoid backflow of the solution. Then, the wound was closed, and the animals were observed until fully recovered from anesthesia.

### Immunofluorescence

Coronal plane microtome-cut Sects. (5 μm) (Thermo Scientifc HM 325) spanning the SNpc and striatum were processed by immunofluorescence for the detection of TH + cells. Twelve sections of each rat were used for immunological staining and cell counting in ipsilateral SNpc. SNpc area was delimited according to the anatomical coordinates from the Atlas of rat brain by Paxinos and Watson (2013). The slices used for SNpc analysis were conducted in the anterior–posterior anatomical coordinates in bregma Point 1: anteroposterior + 0.5 mm; mediolateral − 2.5 mm; dorsoventral − 5.0 mm. Point 2: anteroposterior − 0.5 mm; mediolateral − 3.0 mm; dorsoventral − 6.0 mm. Point 3: anteroposterior − 0.9 mm; mediolateral − 3.7 mm; dorsoventral − 6.5 mm. The paraffin sections were dewaxed in xylene, rehydrated in a gradient of ethanol (100%, 95% and 80%) and distilled water. Antigenic recovery was performed in citrate buffer 30º min at 95ºC (10 mM citric acid, pH 6.0). The samples were washed with 0.1 M PBS + 0.5% Triton and blocked with animal free block and diluent (Vector Laboratories, SP-5035) for 60 min. After this period, they were incubated with primary antibody diluted in 0.1 M PBS containing 0.5% Triton and animal free block 1% (Vector Laboratories, SP-5035) overnight at 4ºC. After incubation with the primary antibody, the samples were washed three times with 0.1 M PBS + 0.5% Triton and incubated with VectaFluor Duet Immunofluorescence Double Labeling Kit, Dylight 488 Anti-Rabbit (green)/Dylight 594 Anti-mouse (red) (Vector Laboratories, DK-8818). Finally, after 5 washes with 0.1 M PBS, the samples were assembled using VECTASHIELD Hard SET + DAPI Antifade Mounting Medium, (Vector Laboratories, H1500) and analyzed using a Leica TCS-SP8 confocal microscope.

### Confocal Analysis and Cell Counting

A series of optical sections with 0.5-µm intervals was analyzed to determine an upper and lower threshold using the Z/Y position for the Spatial Image Series setting using a Leica TCS-SP8 confocal microscope (SACE, University of Murcia). The optical series covered a 5-μm thick slice of tissue. For high magnification, × 20 and × 63 glycerol immersion objectives were used with 0.75 optical zoom (x-axis and y-axis, 1024 × 1024 pixels). The fluorescence measurement or quantification of cells marked for TH was performed in a 20X or 63X objective. To avoid quantification and measurement errors, all images were obtained under the same configuration conditions. For the quantification of TH immunopositive cells, we used the ImageJ software (NIH, Bethesda, MD, United States).

### Quantification of TH-Positive (TH +)

In SNpc, the total number of TH + cells was determined using the 20 × objective and results are expressed as the number of TH + cells. The counting unit was the nucleus surrounded by the immunoreactive cytoplasm. The number of TH immunoreactive neurons was presented as the percentage of TH + cells considering the negative control group as 100%.

### Histology

One centimeter (1 cm) long segments of the ileum and colon of each rat were collected, washed and fixed for 4 h in buffered 4% paraformaldehyde (PFA). After fixation, the segments were submitted to histological routine to perform histomorphometric, quantitative and histopathological analyses. The segments were dehydrated in ascending series of ethanol (70°, 80°, 90° and 99.6°), diaphanized in xylene and embedded in paraffin to obtain semi-serial cross-sections with 4 μm thickness. From each intestinal segment of each animal, 4 sections were placed under glass slides (26 × 76 × 1.2 mm) with a frosted tip. Afterwards, the sections were deparaffinized in xylene, rehydrated in descending series of ethanol (99.6°, 90°, 80°, 70°) and stained in hematoxylin and eosin (H&E) for quantitative, histomorphometric and histopathological analysis of the strata that compose the wall of the small and large intestines and for the quantification of intraepithelial lymphocytes. Another 4 sections (from the same intestinal segment) were placed on glass slides, deparaffinized in xylene, rehydrated in descending series of ethanol and Periodic Acid Schiff (PAS) stained to quantify goblet cells that secrete neutral mucins and labile sialomucins (Vicentino-Vieira et al. 2017). Then, slides were mounted with 25 μL of Permont® (Fisher Scientific) and coverslip (24 × 50x0.13 mm). Readings were taken 36 h after mounting the slides.

### Morphometric Analysis

Four micrometer -thick (4 µm) sections of the ileum and colon, stained in H&E, were used in the histomorphometric analysis. For that, images were captured with the aid of a digital camera (Olympus® SC30, 3.0 Megapixel) attached to an optical microscope (Olympus® BX43F—Minato-Ku, Japan) and used to perform the measurements. Image Pro Plus 4.5.0.29 software (Media Cybernetics Silver Spring, MD, USA) was used for measurements.

The images captured with the 20 × objective lens were used to measure the thickness of the following parameters: muscular, submucosal and mucosal layers, height and width of villi (ileum), depth and width of crypts. Sixty-four measurements of each parameter were performed, with 16 measurements per quadrant of the ileum and colon of all rats. The width of the villi was obtained from the average of three measurements taken at the base, middle third and apex (Vicentino-Vieira et al. 2017).

### Histopathological Analysis

Sections stained in H&E were used for histopathological analysis of the ileum wall and colon of rats. Pathological analysis was performed with the aid of a photonic microscope through objective lenses of 20, 40 or 100x, in 20 microscopic fields per animal. The semiquantitative analysis (scores) of the histopathological findings was performed according to the criteria described by Remke et al. (2024) and Boeing et al. (2021), with the following adaptations.

### Quantitative Analysis of Intraepithelial Lymphocytes (IELs)

Sections stained in H&E were also used for quantification of intraepithelial lymphocytes (IELs). 2560 consecutive ileal and colon epithelial cells from each rat were counted and the IELs present in this interval were counted. In this way, the ratio of IELs/100 epithelial cells for each intestinal segment of each rat was calculated. Quantification was performed directly under a photonic microscope (CX31 Olympus model) with the aid of a 40 × objective lens in fields distributed in alternating quadrants of four different sections for each organ of each rat, thus counting 640 epithelial cells/quadrant/section (Pelaseyed et al. 2014).

### Quantitative Analysis of Paneth Cells

The sections stained in H&E were also used to perform the quantitative analysis of Paneth cells. All Paneth cells present at the base of 64 intestinal crypts present in the mucosa of the ileum of each rat were counted. In addition, the area profile of 64 Paneth cells from each mouse was measured. 16 images, four images/quadrant/slice, were captured using a high-resolution digital camera (Olympus® SC30, 3.0 Megapixel) attached to a trinocular optical microscope (Olympus® BX43F—Minato-Ku, Japan) with the aid of an objective lens. of 40 × and microcomputer. Image analyzes were performed using the Image Pro Plus software (Media Cybernetics) (Vicentino-Vieira et al. 2017; Santos et al. 2018).

### Quantitative Analysis of Goblet Cells

PAS-stained sections were used to quantify goblet cell density in ileal and colonic epithelium. 2560 consecutive epithelial cells were counted in the mucosa of each intestinal segment for all mice. The proportion of PAS + phenotype per 100 epithelial cells was calculated. Quantification was performed directly under a photonic microscope (CX31 Olympus model) with the aid of a 40 × objective lens in fields distributed in alternating quadrants of four different sections for each organ of each rat, therefore, 640 epithelial cells/quadrant/slice were counted (Pelaseyed et al. 2014).

### Fecal Microbiota Analysis

For fecal microbiota analysis, after euthanasia the fecal pellets were collected directly from the distal colon of the animals. The DNA of fecal samples was extracted using the QIAamp DNA Stool Mini (QIAGEN, GmbH, Alemanha), according to the manufacturer’s instructions. The DNA concentration was determined by absorbance at 260 nm and the purity was estimated by determining the A260 to A280 ratio. The measurements were performed using a microvolume NanoDrop ® ND-1000 spectrophotometer (NanoDrop Technologies, Wilmington, DE, USA). The purified DNA was stored at −20 °C until use.

Quantitative Real-time PCR (qPCR) was performed to determine the relative abundance of phyla Firmicutes and Bacteroidetes, family Prevotellacea and Entererobactereacea, and genus *Bifidobacterium* sp. and *Lactobacillus* sp. using group-specific 16S rRNA gene primers for each bacterium (Isogen Life Sciences, Netherlands) (Table 2). A short segment of the 16S rRNA gene (200 bp) was specifically amplified by qPCR, using a conserved 16S rRNA-specific primer pair (Table 2) to determine the total amount of commensal bacteria in the feces. Using the same genomic DNA from each sample, qPCR was completed using group-specific primers for the quantification of bacteria.

**Table 2:** Primers used to quantify bacterial populations by quantitative PCR

Group of bacteria	Oligonucleotide sequence	Product size (bp)	Annealing temp (°c)	Reference
Total bacteria (16S)	F:0.5´ACTCCTACGGGAGGCAGCAG3	200	60	Mujico et al., (2013)
	R: 5´ ATTACCGCGGCTGCTGG 3´			
Phylum Firmicutes	F:ATGTGGTTTAATTCGAAGCA	126	60	Murri et al., (2013)/Queipo-Ortuño, et al., (2012)
	R:AGCTGACGACAACCATGCAC			
Phylum Bacteroidetes	F: ´CATGTGGTTTAATTCGATGAT 3´	126	60	Murri et al., (2013)/Queipo-Ortuño, et al., (2012)
	R: 5´AGCTGACGACAACCATGCAG 3´			
Prevotellacea	g-Prevo-F CACRGTAAACGATGGATGCC	527–529	55	Matsuki, et al. (2002)
	g-Prevo-R GGTCGGGTTGCAGACC			
Entererobactereacea	Eco1457FCATTGACGTTACCCGCAGAAGAAGC	195	63	Stalder, et al. (2019)
	Eco1652-R: CTCTACGAGACTCAAGCTTGC			

*F* Forward, *R* Reverse

The reactions were performed in the thermocycler 7500 Fast Real-time PCR System (Applied Biosystems, USA). Each reaction mixture of 10 μL was composed of Master Mix Green SYBR (Applied Biosystems), 0.4 μL of each of the specific primers (forward and reverse) at a concentration of 10 μmol/L and 2 μL of DNA template. The concentration of 2 ng/µL DNA was used in all reactions and the reaction volume was adjusted with MiliQ water to a final volume of 10 μL per capillary according to the manufacturer's manual. Analyses were performed in duplicate.

Amplifications were made with the following temperature profiles: one cycle at 95 °C (2 min), 30 cycles of denaturation at 95 °C (15 s), primer annealing 60 °C (1 min) and one final cycle at 95 °C (15 s). Melting curves were obtained by slow heating from 65 to 95 °C, with fluorescence measurements taken at every 1 oC increase in temperature. A standard curve was generated for each set of primers, and the efficiency of each reaction was determined. Relative quantification was calculated by the 2 ^−ΔΔ^ Ct method (Livak and Schmittgen, 2001). The results are expressed in fold change over control.

#### Statistical Analysis

The results were submitted to the D´Agostino-Pearson test to verify the type of distribution. Normally distributed data were compared using One-way ANOVA followed by Tukey's post-test and presented as mean ± standard deviation. Normally distributed data were compared using the Kruskal–Wallis test and Dunnett's post-test and presented as median ± interquartile deviation. All tests were performed using the GraphPad Prism program (Version 5.01). Data were considered statistically significant when *P* < 0.05.

## Discussion

The ENS is a complex neural network embedded in the intestinal wall that orchestrates the gut's reflex behaviors, playing an essential role in the regulation of many gastrointestinal functions, including motility and fluid secretion. The involvement of the ENS has been shown to be important in the neurodegenerative process underlying PD. PD progresses slowly and many of its signs and symptoms occur when 50–60% of dopaminergic neurons are lost (Furness et al. 2014; Luesma et al. 2024; Das and Sharma 2016). Intrastriatal injection of 6-OHDA was able to reduce the number of TH-positive cells resulting in selective degeneration of dopaminergic neurons mimicking damage caused by PD. 6-OHDA is a neurotoxin that shares structural similarities with dopamine, exhibiting a high affinity for its transporters, consequently, 6-OHDA can enter dopaminergic neurons and cause dysfunction of the same, leading to neuronal death (Paxinos and Watson 2013; Varešlija et al. 2020).

The present study showed that morphological changes occurred in the thickness of the musculature of parkinsonian rats and the treatment with rutin was able to preserve this musculature. It is also possible to observe a reduction in the height of the villi and in the depth and width of the crypts in animals injected with 6-OHDA and treated concomitantly with rutin. The villi are projections in the intestinal lumen that increase the absorption capacity of several molecules, a high rate of cell renewal, which may indicate a reduction in the epithelial surface that was in contact with the intestinal lumen as a protection mechanism (Vicentino-Vieira et al. 2017).

The animals treated with rutin, either alone or in combination with 6-OHDA, showed a reduction in inflammatory infiltrate compared to the control group. Consistently, a decrease in the number of intraepithelial lymphocytes (IELs) was also observed in the ileum and colon of these groups, whereas the 6-OHDA group exhibited an increase in IELs relative to controls. The intestinal epithelium is constantly exposed to an abundance of antigenic stimuli derived from commensals, food particles, and pathogens present in the intestinal lumen (Nazmi et al. 2021). IELs are a large and diverse population of immune cells residing among epithelial cells that form the intestinal mucosal barrier. Although there are many theories about their physiological role, these cells are known to contribute to intestinal immune tolerance and the regulation of mucosal immunity. In the intestinal epithelium, IELs are in close contact with enterocytes, particularly in the small intestine, and can be activated in response to various pathological conditions (Pelaseyed et al. 2014; Ma et al. 2021).

Among other mechanisms of intestinal cellular defenses, there is the existence of Paneth cells that are part of the innate mucosal immune system in the intestine and play an important role in the innate intestinal defense system, releasing substances stored within their granules. Although the mechanisms that drive this process are not understood, some studies suggest that the number of Paneth cells increases after intestinal damage and may reflect a compensatory intestinal defense response. In this work we observed a significant amount of Paneth cells per intestinal crypt in the ileum of animals injected with 6-OHDA. A recent study describes Paneth cells as key components of the intestinal stem cell niche. In addition to secreting antimicrobial peptides, such as lysozyme and defensins, these cells directly regulate the functional state of intestinal stem cells. The work also shows that aging reduces the synthesis of α-lipoic acid in the intestine, and this decline contributes to the accumulation of abnormal Paneth cells, compromising epithelial homeostasis (Zhang et al. 2025). It was also possible to verify that the treatment with rutin is able to induce the increase and amount of hyperplasia of these cells. Another finding observed was the decrease in goblet cells in the animals injected with 6-OHDA both in the ileum segment and in the colon segment. We observed a decrease in the number of goblet cells, possibly reflecting modulation of the inflammatory response. Goblet cells are producers of protective mucus gel composed predominantly of mucin glycoproteins. Release of mucin into the intestinal lumen provides lubrication and protection against pathogens (Vicentino-Vieira et al. 2017; Gustafsson and Johansson 2022). Goblet cells play a central role in regulating the interaction between the host and the gut microbiota by synthesizing, glycosylating, and secreting the mucin MUC2, a glycoprotein, that forms the structural basis of the mucus layers. The organization and glycidic composition of this mucus, determined by the glycosylation machinery of goblet cells, shape the microbial niche by controlling both the accessibility of bacteria to the epithelium and the availability of glycans that can be used as a substrate by specialized commensal species. Thus, goblet cells not only establish the physical barrier that limits bacterial approach but also influence microbial selection and intestinal homeostasis. Alterations in the function of these cells or in the integrity of the mucus they produce can compromise the segregation between microbiota and epithelium, favoring dysbiosis and contributing to inflammatory bowel diseases (Luis and Hansson 2023). The gut microbiota communicates with epithelial cells, including goblet cells and Paneth cells, and immune cells in the lamina propria, which in turn communicate with neurons and enteric glial cells and nerve endings to modulate the local reflex arc and control gut function (Sharkey and Mawe 2023; Scheperjans et al. 2015).

Recent research has demonstrated the existence of complex communication between the gut and the brain, establishing a potential route for the spread of pathological processes involved in Parkinson's disease (PD). Growing evidence supports the hypothesis that the gut microbiota plays a central role in this bidirectional communication, influencing mechanisms associated with the pathogenesis of PD (Socała et al. 2021; Salim et al. 2022). The human microbiota, acquired at birth and developed concurrently with the host, is an essential component of the organism and actively participates in the regulation of multiple physiological systems, exerting a direct influence on the immune, endocrine, and nervous systems. Its composition is dynamic in nature, being modulated by intrinsic factors, such as age, and extrinsic factors, such as diet, antibiotic use, and environmental conditions (Adak and Khan 2019). The gut microbiota (GM) can influence neurodevelopment, modulate behaviour, and contribute to the onset of neurological disorders (Sampson et al. 2016; Socała et al. 2021; Forsythe et al. 2012). Several studies have demonstrated changes in the abundance of certain bacterial groups in individuals with PD and other neurodegenerative diseases. Lin et al. (2018) observed an increase in the relative abundance of potentially pro-inflammatory bacteria belonging to the Enterobacteriaceae family, which may contribute to inflammatory processes and immune activation. These findings corroborate the results described by Scheperjans et al. (2015), who also reported an increase in this bacterial family in patients with postural instability and gait difficulties, when compared to those with a predominant form of tremor in PD. In the same study, a reduction of approximately 77% in bacteria of the Prevotellaceae family was reported in individuals with PD. It is known that dysbiosis, characterised by changes in the composition and abundance of these intestinal microorganisms, can affect both the enteric nervous system and the central nervous system (CNS). Furthermore, the presence of dysbiosis has been described years before the appearance of the first motor signs in these patients (Sun and Shen 2018). A systematic review aimed at examining the effectiveness of probiotics in the context of neurodegenerative diseases highlights the importance of *Lactobacillus* and *Bifidobacterium* strains for their beneficial effects in neurodegenerative diseases (Socała et al. 2021). These bacteria, by modulating the gut microbiota, can reduce inflammation and oxidative stress, improve mitochondrial function, and influence communication in the gut-brain axis. In vivo and clinical studies indicate that probiotics containing *Lactobacillus* contribute to slowing the progression of disorders such as PD, promoting neuroprotective effects through the regulation of immunological and metabolic processes (Ojha et al. 2023). Studies have demonstrated significant differences in the composition of the gut microbiota of patients with PD (Yan et al. 2021). In analyses of faecal samples, a greater abundance of the genera *Alistipes, Bifidobacterium,* and *Parabacteroides* was observed, as well as a reduction in Faecalibacterium, a group recognized for its production of short-chain fatty acids (SCFA) (Zapała et al. 2021). A higher prevalence of the phylum Bacteroidetes, the class Corynebacteria belonging to the phylum Actinobacteria, the class Deltaproteobacteria of the phylum Proteobacteria, and the genera *Butyricimonas, Robinsoniella,* and *Flavonifractor* were also found. Moreover, the species *Akkermansia muciniphila*, *Eubacterium biforme*, and *Parabacteroides merdae* were identified as more frequent in the gut microbiota of individuals with PD (Zapała et al. 2021; Chen et al. 2022), and further studies could be conducted to characterize other microbial composition that can be affected with the flavonoid treatment in PD models.

Modified physiological functions in individuals affected with PD, such as altered intestinal absorption, reduced gastric motility or eating habits, represent factors that can change the GM ing dysbiosis in these patients (Sampson et al. 2016). Flavonoids have important activities on GM and studies show that flavonoids, including rutin, have modulatory activities on the GM balance, favoring the balance of the defense system and influencing intestinal health (Power et al. 2016). Parkar et al. 2013 have observed that the fermentation of polyphenols stimulated the proliferation of bifidobacteria and decreased the ratio of Firmicutes to Bacteroidetes Mansoorian (2019) (Mansoorian et al. 2019) described that the use of polyphenols helps in the prevention of colorectal cancer. However, to date, there are no studies in literature that demonstrate the influence of the flavonoid rutin on the GM in PD.

## Conclusion

The findings of this study demonstrate that 6-OHDA administration induces significant structural changes in the intestine, ranging from modifications in muscle thickness and villi morphometry to imbalances in the immune elements of the mucosa. These changes reflect a state of intestinal dysfunction associated with neurodegenerative damage, corroborating the hypothesis that the gastrointestinal tract plays a central role in the pathophysiology of Parkinson's disease. The treatment with rutin has been shown to attenuate some of these changes, preserving intestinal morphological integrity, reducing inflammatory infiltrate, and modulating relevant components of the mucosal immune defence, suggesting a potential protective effect. In addition, the data discussed reinforce the importance of the intestinal microbiota in bidirectional gut-brain communication. In this context, natural compounds with antioxidant properties and modulatory potential, such as rutin, emerge as promising alternatives in the search for interventions capable of acting simultaneously on the enteric nervous system, the intestinal immune response, and microbial balance. The results of this study expand our knowledge of the intestinal mechanisms involved in PD and suggest that rutin may play a relevant role in modulating inflammatory and structural processes associated with the disease. However, further studies are needed to fully elucidate the mechanisms of action of rutin and its therapeutic potential in the context of neurodegeneration in PD.

## Data Availability

No datasets were generated or analysed during the current study.
